# Online training course on critical appraisal for nurses: adaptation and assessment

**DOI:** 10.1186/1472-6920-14-136

**Published:** 2014-07-05

**Authors:** Eva Reviriego, María Ángeles Cidoncha, José Asua, Marie Pierre Gagnon, Maider Mateos, Lucía Gárate, Elena de Lorenzo, Rosa María González

**Affiliations:** 1 Researcher, Basque Office for Health Technology Assessment, Ministry for Health, Basque Government, Vitoria-Gasteiz, Spain; 2 Lecturer and Nursing Researcher in the Basque Health Service-Osakidetza, Vitoria-Gasteiz, Spain; 3 Head of Basque Office for Health Technology Assessment, Ministry for Health, Basque Government, Vitoria-Gasteiz, Spain; 4 Associate Professor. Faculty of Nursing Sciences, Université Laval, Québec, Research Centre of the Centre Hospitalier Universitaire de Québec, Québec, Canada; 5 Nursing Research Supervisor, Araba University Hospital, Basque Health Service-Osakidetza, Vitoria-Gasteiz, Spain; 6 Associate Professor, School of Nursing of Vitoria-Gasteiz, Vitoria-Gasteiz, Spain; 7 Subdivision of Quality, Office for the Strategy of Chronicity, Basque Health Service-Osakidetza, Vitoria-Gasteiz, Spain

**Keywords:** Evidence-based practice, Critical appraisal, Nursing, E-learning

## Abstract

**Background:**

Research is an essential activity for improving quality and efficiency in healthcare. The **objective** of this study was to train nurses from the public Basque Health Service (Osakidetza) in critical appraisal, promoting continuous training and the use of research in clinical practice.

**Methods:**

This was a prospective pre-post test study. The InfoCritique course on critical appraisal was translated and adapted. A sample of 50 nurses and 3 tutors was recruited. Educational strategies and assessment instruments were established for the course. A course website was created that contained contact details of the teaching team and coordinator, as well as a course handbook and videos introducing the course. Assessment comprised the administration of questionnaires before and after the course, in order to explore the main intervention outcomes: knowledge acquired and self-learning readiness. Satisfaction was also measured at the end of the course.

**Results:**

Of the 50 health professionals recruited, 3 did not complete the course for personal or work-related reasons. The mean score on the pre-course knowledge questionnaire was 70.5 out of 100, with a standard deviation of 11.96. In general, participants’ performance on the knowledge questionnaire improved after the course, as reflected in the notable increase of the mean score, to 86.6, with a standard deviation of 10.00. Further, analyses confirmed statistically significant differences between pre- and post-course results (p < 0.001). With regard to self-learning readiness, after the course, participants reported a greater readiness and ability for self-directed learning. Lastly, in terms of level of satisfaction with the course, the mean score was 7 out of 10.

**Conclusions:**

Participants significantly improved their knowledge score and self-directed learning readiness after the educational intervention, and they were overall satisfied with the course. For the health system and nursing professionals, this type of course has the potential to provide methodological tools for research, promote a research culture, and encourage critical thinking for evidence-based decision making.

## Background

Research is an essential activity for the advancement of health sciences, and research results should contribute to improving quality and efficiency in healthcare. Evidence-based clinical practice implies that decision making is based on the critical and conscious use of the most recent research results. In their practice, healthcare professionals should also consider the preferences and values of patients, personal and clinical experience and available resources [1-5].

A common strategy for updating knowledge on a given clinical problem, as the basis for changing clinical practice, is to conduct a systematic review of the literature. Such systematic literature reviews allow us to obtain and synthesise the information necessary to support decision making [6]. The assessment of the scientific evidence, however, poses a challenge for nurses: they must be conscious of potential bias in published literature and develop skills for critical appraisal to determine whether or not to trust articles they read on a given heath issue [7-10]. A lack of training in research has been identified as one of the most important barriers to the use of research findings in nursing practice [11-15].

The Internet is increasingly used as a platform for learning in health sciences-related disciplines. Thanks to the success of online self-learning resources, the public sector, universities and businesses all recognise the importance of self-learning abilities, as a necessary workplace skill in the 21st century [16-20]. Indeed, self-directed learning, the autonomous activity of students by which they take on responsibility for their own training, is widely considered the most suitable approach to ensure that professionals are up-to-date and informed about the scientific literature relevant to their professional practice. Accordingly, self-directed learning has been recommended to achieve effective and efficient training of various health professionals [21]. In nursing self-directed learning has been shown to increase knowledge, positive attitudes and behaviour related to evidence based practice [22,23].

Increasing numbers of courses for professional training are available, responding to the need of health professionals to update their knowledge and acquire skills, to continually inform their practice based on scientific evidence and to adapt to new performance requirements. Virtual education, also called e-learning, allows students to choose when to study, this being a good option for motivated individuals who work and want to study during their spare time; it is an excellent tool to help users learn new concepts and consolidate knowledge and skills, thus increasing the independence and motivation of students [24-29].

Given the lack of research culture in nursing and that a lack of skills in this area is a notable main barrier to evidence-based practice, the Ministry for Health of the Government of the Basque Country considered it a priority to develop an online training course to strengthen the relationship between research and clinical practice among nurses.

The general aim of this study was to train nurses from the public Basque Health Service (Osakidetza) in critical appraisal, promoting continuous training and the use of research in clinical practice.

The specific objectives included: 1) to increase knowledge of nurses in the use of critical appraisal skills for use of research; 2) to assess readiness for the use of e-learning as a learning platform; and 3) to investigate the satisfaction with the course, both its content and methods.

## Methods

This was a prospective pre-post study. In order to determine the level of association between the educational intervention and the study variables, questionnaires were administered before and after the critical appraisal course.

### Assessment of e-learning education programs of critical appraisal

A literature search was conducted on educational interventions for critical appraisal skills. Laval University offers educational courses on this subject for different health disciplines including medicine and nursing. Specifically, the InfoCritique program trains students in critical appraisal of scientific literature, with assessments based on tests about the content of each course module.

Our research team evaluated the contents and usefulness of InfoCritique with a view to adapting it to our setting.

### Translation and cultural adaptation of InfoCritique

The InfoCritique program was originally developed in French at Laval University (Quebec, Canada) by the Faculty of Medicine and adapted for nursing by the Faculty of Nursing [30]. A team of two health professionals from the Universidad del Valle in Colombia, both bilingual with Spanish as their mother tongue and with experience of working with scientific literature, translated the materials of the InfoCritique platform from French to Spanish, and coordinated with a team from the University of the Basque Country for the adaptation of all the theoretical models and case studies into Spanish.

This translation was then adapted to Castilian Spanish in order to make it easier to understand by the target population, checking whether the vocabulary was suitable and the items were culturally applicable. We also assessed the conceptual equivalence and clarity of each of the course modules.

The translation and adaptation process of the Infocritique modules was made following the various stages of cross-cultural validation and has been described in detail elsewhere ref. [31].

### Study population

The target population was composed of nurses from different areas of the Basque Health Service-Osakidetza (primary care, specialised care and mental health) with basic knowledge of research methodology. Participants were selected through various Departments of Nursing, requesting their collaboration to identify working nurses with an academic degree in Nursing who had a basic knowledge of research and were interested in learning more about it and willing to participate in the educational intervention.

### Sample size

The sample size for this study was determined by the number of tutors (three) available from the research team to monitor the educational intervention and by the number of nurses interested in participating who met the inclusion criteria and were therefore eligible to be recruited. As a consequence, we did not calculate the sample size a priori; rather we used a convenience sample size of 50 nurses. Participation in the study was voluntary, and participants received continuing education credits for completing modules.

### Description of the intervention

The InfoCritique program trains students in the critical appraisal of scientific literature and assesses them based on tests focusing on the content of each module. The four self-directed learning modules available in Spanish cover 1) clinical trials; 2) systematic reviews; 3) diagnostic tests; and 4) qualitative studies.

Every module begins with some basic information about the type of research study under consideration and associated methods. Then, there are one or more exercises focused around a clinical case. For each clinical case, students are presented with a scientific article reporting a study of the design covered in the module. Students have to read the article and use a critical appraisal grid adapted to each study design in order to appraise the study. Each module also provides interactive links to supplementary information, such as a glossary and additional references. Finally, students have to complete an assessment test and a satisfaction form for each module, which is used as feedback for the tutors [32].

The 50 nurses recruited had to take the aforementioned 4 modules of online training related to critical appraisal skills. They were informed that each module required 6 hours of student work. It was explained that the platform had online content with interactive and dynamic assessments that must be passed to obtain the official diploma for the course.

Throughout the process, the participants could receive methodological support through the computer program itself and via e-mail. The three tutors were responsible for approving the course content, answering questions, and monitoring progress of students through the course, and compiling a report on students’ questions after completion of the course. The nurses were assessed before and after the educational intervention to determine the impact of the course on a specific list of outcome measures.

A course website http://infocritique.wordpress.com/ was created to support students throughout the course, with information about the teaching methods and how to directly access the Spanish InfoCritique educational platform. In addition, the website provides the following: a main page introducing the course, a page with contact details of the team coordinating the course and tutors, introductory videos from all the institutions involved, and a course handbook with information about the objectives, practicalities and assessment of the course.

### Outcome measures

The following three instruments were used for evaluating the online training pilot (for translations of corresponding questionnaires see Additional files 1, 2, 3):

– Skills acquired, assessed using a **knowledge** assessment questionnaire administered before and after taking the online training course. For this, an *ad hoc* questionnaire was drawn up with 20 questions to test students’ knowledge of the contents of the training course. These 20 questions were randomly selected from the final assessment tests included in each of the four modules. From each module, five questions were incorporated.

– **Self-learning ability,** using the Self-Directed Learning Readiness Scale for Nursing Education (SDLRSNE) [33-35]. We obtained permission from Fisher, King and Tague (2001) to translate the Self-Directed Learning Readiness Scale [33]. The translation was then made into Spanish consulting both the original version in English and the French version used at Laval University. The questionnaire, composed of 40 items divided into 3 subscales, Self-management (13 items), Desire for learning (12 items) and Self-control (15 items), was administered before and after the course. In this research, the self-directed learning readiness was measured using the 40 items of the SDLRSNE rated on 5-point Likert scales ranging from strongly disagree to strongly agree. The Spanish version of the SDLRSNE scale was validated following the steps proposed by Vallerand for transcultural validation [36].

– **Satisfaction,** measured in two ways.

– After finishing each module, with a structured questionnaire composed of 10 closed questions plus a space for comments on the contents, platform and time spent.

– At the end of the programme, with an *ad hoc* satisfaction questionnaire, composed of 20 items to assess the following factors: practicalities of the course and the methodology, content of the modules, characteristics of the website and overall impression.

A specific questionnaire was also designed to collect data on participants’ socio-demographic characteristics, covering gender, age, year of completion of their first nursing diploma, level of education, number of hours devoted to research training, whether they put into practice methodological research knowledge, and current position (Additional file 4).

### Method for distributing the questionnaires

The aforementioned assessments were combined into two batteries of questionnaires, one administered before and one after completing the course. Specifically, before the course, nurses were asked to complete two questionnaires: the knowledge questionnaire and the SDLRSNE; and after the course, they were asked to repeat the same two questionnaires and, in addition, to fill out a final satisfaction questionnaire. Note that this last questionnaire also included open-ended questions allowing participants to comment on various aspects of the modules.

The questionnaires were implemented using an online survey management system that ensured the confidentiality of the responses. A personalised link was sent to each of the 50 participants, explaining the purpose of the questionnaires and indicating the deadline for submitting their responses. Two reminders were sent to request completion of these questionnaires.

### Data analysis

For each of the outcome measures we performed two-stage statistical analysis:

– Description of the data: A descriptive analysis of the responses to each of the questionnaire items before and after the intervention was conducted. Measures of central tendency (mean and median) and dispersion (standard deviation) were calculated for quantitative data and frequencies/percentages for qualitative variables. In both cases, the presentation of the results includes graphical representations to help visualise the data.

– Pre-post comparison: To assess whether there were statistically significant differences before and after the course the following tests were performed in accordance with the nature of the variables: for qualitative variables, the McNemar test; and for quantitative variables, a Student’s t-test for paired samples or the Wilcoxon tests depending on whether data were normally distributed or not. Normality was tested using the Shapiro-Wilk test. The confidence level was set at 95%.

In the case of the SDLRSNE score, we measured the internal consistency of the instrument using Cronbach’s alpha both for the overall questionnaire and for its three subscales (Self-management, Desire for learning and Self-control).

Finally, univariate general linear models were built to assess the association of some variables, such as previous training and computer skills, with pre-post course changes in scores on the knowledge questionnaire, and with satisfaction scores. Specifically, these models involved hypothesis testing for the correlation coefficient when the independent variable was quantitative and one-factor ANOVA when it was qualitative.

All the statistical analyses were performed using IBM SPSS Statistics (version 19).

### Ethical considerations

Nurses participated in the study voluntarily. It was understood that they had agreed to participate once they had been told about the objectives of the study and what participation would involve, given written approval and completed the pre-course questionnaire.

Abandonment of the study was not penalised in any way, and the identity of any individuals who dropped out was kept confidential. Further, steps were taken to safeguard the anonymity of participants and confidentiality of the data, throughout the process. Specifically, codes were used in place of identifying data and it was ensured that samples of text taken from answers to open questions did not include information that might help to identify the corresponding participant. Indeed, all the information related to the study was kept confidential, only members of the research team having access to it. Ethical approval was not required given that the study was an evaluation of a learning activity.

## Results

### Socio-demographic

Of the 50 people who started the course, 46 were women and 4 men. This sex ratio in the sample (92% *vs.* 8%) can be attributed to greater representation of women in health professions in Spain, especially in nursing. In 2011 (the most recent data from the Spanish National Statistics Institute), the college of health professionals with highest percentage of women was that for nurses (84.0%, the figure for midwifes being 93.9%) followed by pharmacists (70.0%) (INE 2011). The distribution of participants by age group was relatively balanced, over a third of participants (38%) being between 41 and 50 years of age. All the nurses had an academic degree and 84% of the sample was working in a clinical setting, 12% in management and 4% in teaching.

Of the 50 people who started the course, 3 did not finish it for personal or work-related reasons. These three people were women aged between 50 and 54 years old who reported in the pre-course questionnaire that they had very little experience of research-related training.

### Knowledge

**Figure 1:**
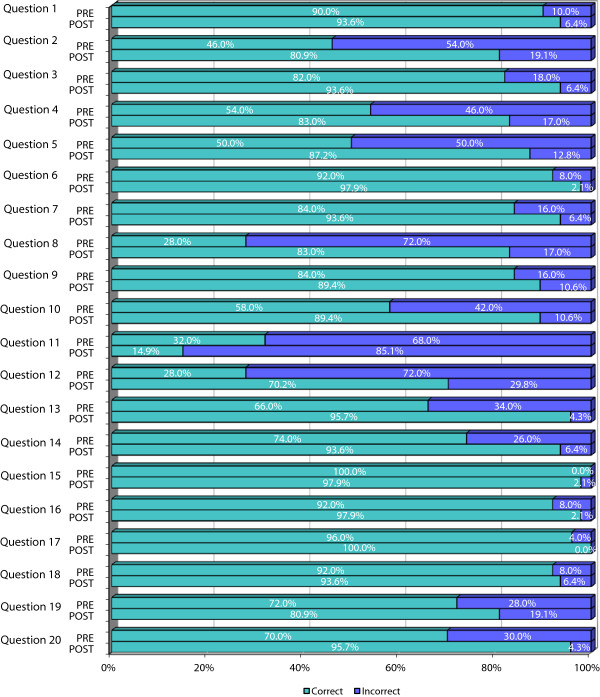
Comparison of pre- and post-course knowledge questionnaires results.

Figure 1 combines the results obtained for each of the 20 questions on the knowledge questionnaire before and after the course, facilitating the comparison and visualisation of the changes. First, it is clear that there were issues about which participants already had considerable knowledge prior to the study. In particular, more than 90% of them gave correct answers to questions such as “In a systematic review, researchers should always try to identify differences between the studies considered” (item 6), “The sample size is important in the evaluation of the validity of a test” (item 15), and items related to qualitative research (items 16, 17 and 18). In other areas, however, less than half of the participants gave correct answers. For instance, they had pre-course gaps in their knowledge for questions 2, 8, 11 and 12 related to epidemiological concepts (significance or sensitivity and specificity).

Second, the figure also clearly illustrates that the nurses’ results improved. In fact, after the course, only one question (question 11: sensibility and specificity) was answered incorrectly by more than 30% of participants. In this question, the percentage of correct answers was very low (14.9%), and this made the research team wonder whether the question itself might have been confusing or the related content insufficiently clear in the course.

The mean score on the knowledge questionnaire increased from 70.5 out of 100 (SD 11.96) pre-course to 86.6 (SD of 10.00), and this improvement was statistically significant (p < 0.001). Analysing the results item by item, it was found that the questions for which there were statistically significant pre- and post-differences were precisely those in which there were the most errors in the initial assessment, that is, those with the greatest margin for improvement (Table 1).

**Table 1:** Comparison of pre- and post-course knowledge questionnaire scores (McNemar test)

	**Statistic**	**p-value**		**Statistic**	**p-value**
**Question 1**	0.167	0.687	**Question 11**	3.857	0.078
**Question 2**	12.5	< 0.001*	**Question 12**	17.391	< 0.001*
**Question 3**	3.125	0.07	**Question 13**	12.071	< 0.001*
**Question 4**	9.6	0.001*	**Question 14**	6.75	0.006*
**Question 5**	13.47	< 0.001*	**Question 15**	---	---
**Question 6**	0.8	0.375	**Question 16**	0.5	0.5
**Question 7**	0.8	0.375	**Question 17**	---	---
**Question 8**	12.5	< 0.001*	**Question 18**	0.25	1
**Question 9**	0.8	0.375	**Question 19**	1.067	0.302
**Question 10**	10.56	0.001*	**Question 20**	7.69	0.003*

*The number of correct answers increased significantly after the course.

### Self-directed learning ability

#### *Reliability*

**Figure 2:**
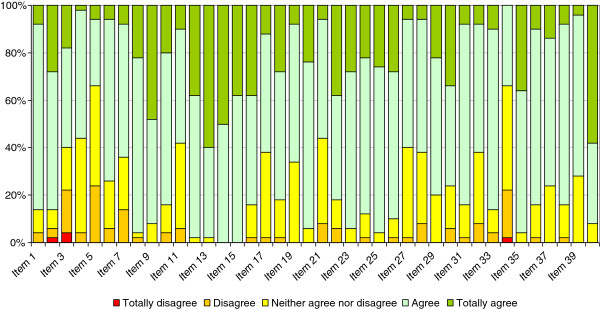
Results of the pre-course Self-Directed Learning Readiness Scale for Nursing Education (SDLRSNE).

**Figure 3:**
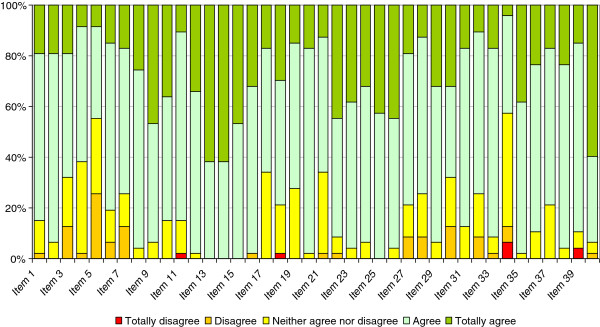
Results of the post-course Self-Directed Learning Readiness Scale for Nursing Education (SDLRSNE).

The Cronbach’s alpha for the complete questionnaire was 0.884, confirming the internal consistency of the scale. The coefficients for each of the subscales of the instrument were also high: 0.821 for Self-management, 0.812 for Desire for learning and 0.742 for Self-control. We confirmed that these coefficients were not very sensitive to the removal of single items from the subscales, the value of Cronbach’s alpha remaining stable after removing one of the questions.As can be observed in Figure 2, there was a high readiness for self-directed learning before the course, the options “agree” and “totally agree” being selected by most participants for most of the items on the scale. Only for items 5 and 34 were one of these two response options selected by less than 50% of participants. The total scores ranged from 136 to 183 out of 200, with a mean of 157.92 (SD: 14.48), reflecting the overall good level of readiness for self-directed learning among participants at baseline.As shown in Figure 3, the pattern of post-course answers was very similar to that observed before the course, but on most items there was a slight increase in the percentage of participants selecting “totally agree”. The mean post-course score was 163.06 (SD 13.71), more than 5 points higher than the pre-course score, and this difference was statistically significant (Student’s t-test for paired samples, p < 0.001). These results indicate that after the course participants felt more empowered and had an even greater readiness for self-directed learning.

### Satisfaction

#### *Participation and contents*

**Figure 4:**
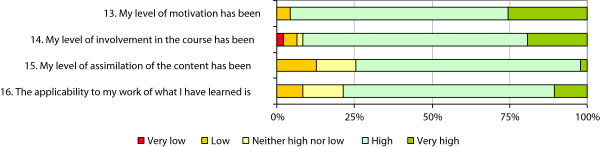
Participation in the course.

**Figure 5:**
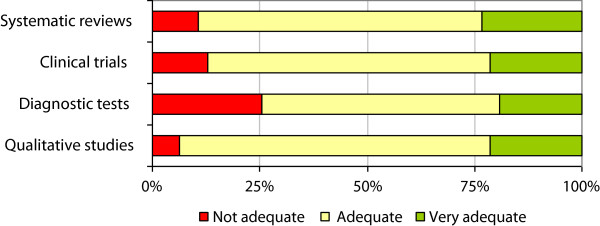
Rating of content of the course.

The satisfaction questionnaire results (Figure 4) showed that the response to the course was very good in terms of variables related to participation. In general, participants reported good or very good levels of motivation and commitment. On the other hand, 25% of them recognised that they had not assimilated the content very well (low to moderate rating).In general, the four modules of the course were considered to be adequate or very adequate in terms of content (Figure 5). Notably, however, a quarter of participants considered that the diagnostic tests module was not adequate.

We detected some difficulties related to continuing with the course due to insufficient pre-course knowledge, especially with regards to statistics. Although not many of the participants would have added new modules, several areas such as literature searches and types of experimental design were mentioned as possible additional content. Suggestions included the creation of a forum to improve communication between participants as well as between participants and tutors, and more explanations of the statistical concepts.

Despite the module on diagnostic tests being considered the most difficult, it was one of the most popular. The module on qualitative studies was also popular and many participants also appreciated the usefulness of the case reports module.

#### *Characteristics of the website*

The most valued characteristics of the website were that it was simple and user friendly, loaded rapidly, interactive and well structured. On the other hand, the design was considered weak, not being very attractive and lacking a space for interaction between tutors and participants. The great majority of participants thought that navigation of the website was easy and was no obstacle to the practical functioning of the course. Overall, there was a high level of satisfaction with the website.

#### *Overall rating of the course*

At the end of the questionnaire, participants were asked to rate the course overall; the mean obtained was 7, with scores ranging from 2 to the maximum of 10.

## Discussion

This quasi-experimental study demonstrates that the self-directed educational intervention under study was effective in increasing the knowledge and skills of nurses in relation to evidence-based practice. The findings of this study are consistent with other similar studies that have demonstrated that training in evidence-based practice is necessary. They also highlight that such educational interventions should consider not only increasing knowledge and skills, but also how to transfer them into practice in the workplace and have an impact on health outcomes in patients [20,21].

Our assessment of this online course showed that: 1) nurses did significantly improve their overall knowledge score after the educational intervention; 2) their self-assessment suggested a greater readiness and skills for self-directed learning after the course; and 3) they were, in general, satisfied with the course, giving it a rating of 7 out of 10. A high satisfaction rate with web-based education for nurses was also identified in the systematic review of Du et al. [37].

In the post-course questionnaire, participants commented on the usefulness of the course for clinical practice and professional development. These findings are consistent with the three key predictors of satisfaction with web-based training in continuing education discussed in Atreja et al. [38]: effectiveness of the instructional design, website usability and course usefulness.

While some participants found it difficult to put the knowledge acquired into practice, others reported feeling that they were able to transfer this knowledge to colleagues, they had started to question the validity of scientific papers and, by the end of the course, their attitude towards evidence-based practice had changed. On this basis, we conclude that this course does provide useful tools related to research methodology and promote a research culture in nurses, as well as a critical approach to decision making based on scientific evidence.

This study confirms that e-learning is an effective method for continuous training, providing greater independence with regards to organisation of the learning time, as shown in the literature [12-14,23]. On the other hand, in-person training sessions to discuss doubts may be necessary for some learners. Further, several of the participants reported the need for greater tutor-student interaction and proposed the creation of a virtual forum for improving communication between them. In this study, students only had contact with tutors and the coordinating team by email, and this may have limited the interaction between all the agents involved; this might explain the feeling of a need for in-person sessions.

E-learning is recognised as a type of training that gives great independence and flexibility for studying [18], and moreover, this study shows positive results in terms of the knowledge acquired and satisfaction. For these reasons, the implementation of this course is justified from economic and organisational points of view. It is now necessary to develop long-term strategies to ensure that the knowledge and skills acquired for critical appraisal are not lost over time and can be applied in daily practice.

Findings from this study could be transferable to similar nurse populations, but more research should be conducted to evaluate the effects of self-directed course in nurses in other settings. This research also contributes to the knowledge of e-learning in continuing education in nursing by providing a specific method for the evaluation of an web-based learning course.

### Limitations

A first limitation of this pilot study is the lack of control group and random assignment, which limit the strength of conclusions that can be drawn about the the effectiveness of the online self-learning modules on critical appraisal skills. However, the pre-test measures provide a comparative baseline for some of the data.

A second limitation is the small size. We sought to obtain a convenience sample of 50 nurses in our setting. However, three nurses withdrew from the study before completing all modules.

Finally, we did not set a threshold for a meaningful point score change in the SDLRSNE score a priori. In future studies, it would be interesting to establish such criteria to assess whether the differences between pre and post intervention scores are important in practice.

## Conclusions

The results of this study demonstrate that e-learning could be an effective approach for continuous professional development for nurses to increase their knowledge of research methodology, as well as help them develop a critical approach to decision making based on scientific evidence. The success of this learning method was reflected in both the assessment of the online course and the enthusiasm of participants.

The main conclusions in the areas assessed were that nurses significantly improved their knowledge score and self-directed learning readiness after the educational intervention; and they were overall satisfied with the course.

This educational intervention responds to the need for specific training in critical appraisal skills in these healthcare professionals, to emphasize and strengthen the relationship between research and clinical practice and promote a research culture in nursing. The study enabled us to assess whether the course meets these training requirements and identify areas for improvement.

## Competing interests

The authors have no conflicts of interest to declare in relation to this manuscript.

## Authors’ contributions

ER and MAC participated in all aspects of the study, coordinating the different stages of the study. JA and MPG were involved in the conception of the study, and the translation of the learning modules, as well as being in charge of the institutional relationships. EDL, LG and RG contributed to the design of the study and participated in the review of the contents of the course, tutoring students. MM conducted the statistical analysis and participated in the interpretation of the study results and review of documents. All authors read and approved the final manuscript.

## Pre-publication history

The pre-publication history for this paper can be accessed here:

http://www.biomedcentral.com/1472-6920/14/136/prepub

## Supplementary Material

Additional file 1 Knowledge Questionnaire. Click here for file

Additional file 2 Self-Directed Learning Readiness Scale for Nursing Education (SDLRSNE). Click here for file

Additional file 3 Satisfaction Questionnaire. Click here for file

Additional file 4 Socio-demographic Questionnaire. Click here for file
