# Supplementary material for: Online training course on critical appraisal for nurses: adaptation and assessment
Source: BMC Med Educ. 2014 Jul 5;14:136. doi: 10.1186/1472-6920-14-136 (PMC4107575; doi:10.1186/1472-6920-14-136)
Supplement: Additional file 1 — Knowledge Questionnaire. [file 1472-6920-14-136-S1.doc]

Knowledge Questionnaire

-----------------------------------------------------------------------

The aim of this questionnaire is to compare your knowledge before and after the course. Thank you again for taking the time to complete this.

The following is the objective of a review article: “The objective of this review is to assess the risk of urinary infection with the use of sterile gloves versus handwashing alone in patients with spinal lesions who need intermittent bladder catheterisation”

Question 1.- What is the target population?

People with urine infections

People using sterile gloves

People who need intermittent bladder catheterisation

People with spinal lesions who need intermittent bladder catheterisation

This information is not given

Question 2.- What is the intervention or exposure under study?

Urinary infection

The use of sterile gloves

Handwashing

Intermittent bladder catheterisation

This information is not given

Question 3.- What is/are the outcome measure(s)?

Urinary infection

The use of sterile gloves

Handwashing

Intermittent bladder catheterisation

This information is not given

Question 4.- What is the control condition?

Urinary infection

The use of sterile gloves

Handwashing

Intermittent bladder catheterisation

This information is not given

Question 5.- A systematic review should always include a meta-analysis.

True

False

Question 6.- In a systematic review, researchers should always try to identify differences between the studies considered.

True

False

Question 7.- Methods of random allocation are processes in which:

Neither the researcher nor the participants know the group assignment (experimental or control)

All participants have the same probability of being assigned to the experimental or control group, as the allocation is performed using a computer-generated random-number sequence

Participants assigned to one or other of the study groups are comparable

The participants are assigned alternately to one or other of the study groups depending on the day of the week

Question 8.- If a clinical trial shows a statistically significant difference between a new treatment and a placebo (p < 0.05) but this difference is not clinically significant, it can be concluded that:

The new treatment is probably useful

The new treatment is probably not very useful

It is impossible to decide without knowing the power of the study

None of the above is true

Question 9.- Among the following statements related to group allocation which is/are true?

A simple blind study is one in which only the participants know which group they are assigned to

A double blind study is one in which only the researchers do not know which group the participants are assigned to

A simple blind study is one in which only the participants or only the researchers do not know which group the participants are assigned to

A simple blind study is one in which neither the participants nor the researchers know which group the participants are assigned to

Question 10.- Rank the following types of studies in descending order of internal validity of the research designs used: a) before and after study, b) randomised clinical trial, c) descriptive study and d) cohort study

b-d-a-c

d-c-a-b

c-a-d-b

a-b-c-d

Question 11.- When screening for a disease a sensitive test is preferred over one that is specific.

True

False

Question 12.- A patient has a high pre-test risk of 20% of developing pressure ulcers. If we do a test with 100% sensitivity and 100% specificity, what is the probability that the patient develops this type of injury if the test is positive?

0%

20%

50%

80%

100%

Question 13.- The validity of a diagnostic test should be evaluated by comparing its results with those of a test that classifies patients as accurately as possible as having or not having the condition?

True

False

Question 14.- The sensitivity and specificity of a test can vary according to the population from which these values are obtained.

True

False

Question 15.- In a study evaluating the validity of a test, the number of patients included is not very important.

True

False

Question 16.- The same rigour is required in the research process for qualitative and quantitative research regardless of the way it is applied.

True

False

Question 17.- When undertaking qualitative research, the role of the researchers must be clearly defined.

True

False

Question 18.- Triangulation of data increases the credibility of qualitative research.

True

False

Question 19.- Qualitative data analysis involves a deductive approach based on pre-existing theories.

True

False

Question 20.- In qualitative research, the concept of external validity of information is described in terms of transferability.

True

False
