# Supplementary material for: Online training course on critical appraisal for nurses: adaptation and assessment
Source: BMC Med Educ. 2014 Jul 5;14:136. doi: 10.1186/1472-6920-14-136 (PMC4107575; doi:10.1186/1472-6920-14-136)
Supplement: Additional file 2 — Self-Directed Learning Readiness Scale for Nursing Education (SDLRSNE). [file 1472-6920-14-136-S2.doc]

Self-Directed Learning Readiness Scale for Nursing Education (SDLRSNE)

-----------------------------------------------------------------------

Please select just one response for each of the statements below.

|  | 1 | 2 | 3 | 4 | 5 |
| --- | --- | --- | --- | --- | --- |
| 1. I solve problems using a plan |  |  |  |  |  |
| 2. I prioritize my work |  |  |  |  |  |
| 3. I do not manage my time well |  |  |  |  |  |
| 4. I have good management skills |  |  |  |  |  |
| 5. I set strict time frames |  |  |  |  |  |
| 6. I prefer to plan my own learning |  |  |  |  |  |
| 7. I am systematic in my learning |  |  |  |  |  |
| 8. I am able to focus on a problem |  |  |  |  |  |
| 9. I need to know why |  |  |  |  |  |
| 10. I critically evaluate new ideas |  |  |  |  |  |
| 11. I prefer to set my own learning goals |  |  |  |  |  |
| 12. I learn from my mistakes |  |  |  |  |  |
| 13. I am open to new ideas |  |  |  |  |  |
| 14. When presented with a problem I cannot resolve, I will ask for assistance |  |  |  |  |  |
| 15. I am responsible |  |  |  |  |  |
| 16. I like to evaluate what I do |  |  |  |  |  |
| 17. I have high personal expectations |  |  |  |  |  |
| 18. I have high personal standards |  |  |  |  |  |
| 19. I have high beliefs in my abilities |  |  |  |  |  |
| 20. I am aware of my own limitations |  |  |  |  |  |

|  | 1 | 2 | 3 | 4 | 5 |
| --- | --- | --- | --- | --- | --- |
| 21. I am confident in my ability to search out information |  |  |  |  |  |
| 22. I do not enjoy studying |  |  |  |  |  |
| 23. I have a need to learn |  |  |  |  |  |
| 24. I enjoy a challenge |  |  |  |  |  |
| 25. I want to learn new information |  |  |  |  |  |
| 26. I enjoy learning new information |  |  |  |  |  |
| 27. I set specific times for my study |  |  |  |  |  |
| 28. I am self-disciplined |  |  |  |  |  |
| 29. I like to gather the facts before I make a decision |  |  |  |  |  |
| 30. I am disorganized |  |  |  |  |  |
| 31. I am logical |  |  |  |  |  |
| 32. I am methodical |  |  |  |  |  |
| 33. I evaluate my own performance |  |  |  |  |  |
| 34. I prefer to set my own criteria on which to evaluate my performance |  |  |  |  |  |
| 35. I am responsible for my own decisions/actions |  |  |  |  |  |
| 36. I can be trusted to pursue my own learning |  |  |  |  |  |
| 37. I can find out information for myself |  |  |  |  |  |
| 38. I like to decisions for myself |  |  |  |  |  |
| 39. I prefer to set my own goals |  |  |  |  |  |
| 40. I am not in control of my life |  |  |  |  |  |

1. Strongly disagree

2. Disagree

3. Neither agree nor disagree

4. Agree

5. Strongly agree
