# Supplementary material for: Online training course on critical appraisal for nurses: adaptation and assessment
Source: BMC Med Educ. 2014 Jul 5;14:136. doi: 10.1186/1472-6920-14-136 (PMC4107575; doi:10.1186/1472-6920-14-136)
Supplement: Additional file 3 — Satisfaction Questionnaire. [file 1472-6920-14-136-S3.doc]

Satisfaction Questionnaire

-----------------------------------------------------------------------

This questionnaire intends to assess your level of satisfaction with this course and the potential usefulness of its content; accordingly, your opinions are very important to us to improve the training offered in the future.

Please indicate your level of agreement with each of the following statements using the scale provided.

Question 1.- Rate the general characteristics of this course:

|  | Not at all | To a little extent | To some extent | To a great extent | Completely |
| --- | --- | --- | --- | --- | --- |
| 1. My expectations of the course have been met |  |  |  |  |  |
| 2. The content meets the objectives of the course |  |  |  |  |  |
| 3. The content is explored in sufficient depth |  |  |  |  |  |
| 4. The practical exercises are useful |  |  |  |  |  |
| 5. The support material (glossary, bibliography, etc.) is useful. |  |  |  |  |  |
| 6. The theoretical explanations are clear. |  |  |  |  |  |
| 7. Support from the tutor when requested makes the course easier |  |  |  |  |  |
| 8. The content is useful for critical appraisal. |  |  |  |  |  |
| 9. The content is useful for being applied in your clinical practice. |  |  |  |  |  |
| 10. The duration of the course is appropriate. |  |  |  |  |  |
| 11. The methodology suits your learning style. |  |  |  |  |  |
| 12. Overall, the course is good. |  |  |  |  |  |

Question 2.- Rate your participation in this course:

|  | Very low | Low | Neither high nor low | High | Very high |
| --- | --- | --- | --- | --- | --- |
| 13. My level of motivation has been |  |  |  |  |  |
| 14. My level of involvement in the course has been |  |  |  |  |  |
| 15. My level of assimilation of the content has been |  |  |  |  |  |
| 16. The applicability to my work of what I have learned is |  |  |  |  |  |

Please tell what you think about the learning method; the information provided will be used to assess the characteristics of the InfoCritique website:

<http://infocritique.fmed.ulaval.ca/Cours/infocritique/index.aspx>.

We need to assess whether the structure and content of the website is appropriate for our setting. Respond to the questions below considering how this platform could be improved to better meet your learning needs.

Question 3.- What do you think of the presentation of the main page, the one that appears after entering your username and password?

______________________________________________________________________________

Question 4.- What do you think in general of the content of the four modules?

|  | Not adequate | Adequate | Very adequate |
| --- | --- | --- | --- |
| Systematic reviews |  |  |  |
| Clinical trials |  |  |  |
| Diagnostic tests |  |  |  |
| Qualitative studies |  |  |  |

Question 5.- Would you omit any of the modules?

Yes

No

Question 6.- Would you change any of the modules?

Yes

No

Question 7.- Would you add any modules?

Yes

No

Question 8.- Please comment on your responses to the last three question, if you would omit, change or add information to the modules.

______________________________________________________________________________

Question 9.- Which was your favourite section of the modules? Which do you think would be the most useful?

______________________________________________________________________________

Question 10.- Can you suggest any other sections we could include on the website?

______________________________________________________________________________

Question 11.- How much importance do you place on the following characteristics of a website?

|  | Not at all important | Not very important | Important | Very important | Not applicable |
| --- | --- | --- | --- | --- | --- |
| Ease of use |  |  |  |  |  |
| Speed of loading of the content |  |  |  |  |  |
| Variety of content |  |  |  |  |  |
| Quality of content |  |  |  |  |  |
| Whether content has been updated |  |  |  |  |  |
| Attractiveness of the design |  |  |  |  |  |
| Web support |  |  |  |  |  |

Question 12.- Navigating the page is:

Very difficult

Fairly difficult

Fairly easy

Very easy

Question 13.- Overall, how satisfied are you with the website?

Completely satisfied

Somewhat satisfied

Somewhat dissatisfied

Completely dissatisfied

Question 14.- What do you most like about this educational platform from its design to its content?

Answer: _____________________________________________________________________________

Question 15.- What do you like least about this educational platform from its design to its content?

Answer: _____________________________________________________________________________

Question 16.- Do you have any other comments or suggestions to improve the web in terms of design or content?

Answer: _____________________________________________________________________________

Question 17.- Sum up in a sentence what you would say to a colleague who was going to take this course:

Answer: _____________________________________________________________________________

Question 18.- Rate the course on a scale from 0 to 10:

Answer: _____________________________________________________________________________

Question 19.- Approximately how many hours have you spent on each module?

Answer: _____________________________________________________________________________

Question 20.- Does the time you have spent seem appropriate to achieve the objectives of each module?

Yes

No
