# Supplementary material for: Online training course on critical appraisal for nurses: adaptation and assessment
Source: BMC Med Educ. 2014 Jul 5;14:136. doi: 10.1186/1472-6920-14-136 (PMC4107575; doi:10.1186/1472-6920-14-136)
Supplement: Additional file 4 — Socio-demographic Questionnaire. [file 1472-6920-14-136-S4.doc]

Socio-demographic data

-----------------------------------------------------------------------

Question 1.- Sex

Male

Female

Question 2.- Age

Answer: _____________________________________________________________________________

Question 3.- Year in which you completed your nursing course at university:

Answer: _____________________________________________________________________________

Question 4.- Do you have other university qualifications?

Bachelor´s degree

Postgraduate diploma

Master’s degree

Doctoral degree

Other

(Please specify)_______________________________________________________

Question 5.- In the last 5 years, how many hours of research-related training have you received?

Answer: _____________________________________________________________________________

Question 6.- Have you put your knowledge of research methodology into practice?

Yes

No

Question 7.- What is your current professional position?

Answer: _____________________________________________________________________________

Question 8.- Which province do you work in?

Alava

Bizkaia

Gipuzkoa

Other

Question 9.- What is your level of computer skills?

Beginner

Intermediate

Expert

Question 10.- How much experience do you have of online courses?

Beginner

Intermediate

Expert

Question 11.- Any other comments are welcome:

Comments:

______________________________________________________________________________

______________________________________________________________________________

This is the end of the questionnaire.

Thank you very much for taking the time to complete it.
